# Deficiency of HIF-1α enhances influenza A virus replication by promoting autophagy in alveolar type II epithelial cells

**DOI:** 10.1080/22221751.2020.1742585

**Published:** 2020-03-25

**Authors:** Caiqi Zhao, Jie Chen, Lianping Cheng, Kaifeng Xu, Yiyu Yang, Xiao Su

**Affiliations:** a The Joint Center for Infection and Immunity, Guangzhou Institute of Pediatrics, Department of Pediatric Intensive Care Unit, Guangzhou Women and Children’s Medical Center, Guangzhou Medical University, Guangzhou, People’s Republic of China; b Unit of Respiratory Infection and Immunity, CAS Key Laboratory of Molecular Virology & Immunology, Institut Pasteur of Shanghai, Chinese Academy of Sciences, Shanghai, People’s Republic of China; c Department of Respiratory Medicine, Peking Union Medical College Hospital, Chinese Academy of Medical Sciences, Beijing, People’s Republic of China

**Keywords:** Influenza A virus, autophagy, HIF-1α, glycolysis, AMPKα, ULK1

## Abstract

Infection of influenza A virus (IAV) can trigger exaggerated pulmonary inflammation and induce acute lung injury (ALI). Limiting IAV replication and alleviation of pulmonary inflammation are two important therapeutic strategies for influenza virus infection. Recent studies have shown that hypoxia inducible factor-1α (HIF-1α) is an essential factor for the development and repair of ALI; however, the role and the underlying mechanisms of HIF-1α in IAV-induced ALI remain elusive. Here, we demonstrated that lung epithelial cell-specific *Hif1α* knockout mice infected with IAV developed more lung IAV replication and severe lung inflammation, which led to increased mortality compared to IAV-infected control mice. Moreover, knockdown of *HIF1A* in A549 cells (human alveolar type II epithelial cell line) promoted IAV replication *in vitro*. Mechanistically, knockdown of *HIF1A* reduced glycolysis by regulating transcription of glycolysis-related enzymes, which subsequently activated the AMPKα-ULK1 signalling pathway. Interestingly, AMPKα-ULK1 signalling promoted autophagy and augmented IAV replication. Taken together, deficiency of HIF-1α in lung epithelial cells reduces glycolysis and enhances AMPKα-ULK1-mediated autophagy, which finally facilitates IAV replication. These findings have deepened our understanding of the role of HIF-1α in regulating IAV replication and provided us novel therapeutic targets for combating influenza infection.

## Introduction

Influenza A virus (IAV) is an enveloped virus with a segmented negative-sense RNA genome and is classified according to its surface glycoproteins, hemaggluinin (HA) and neuraminidase (NA) [1]. H1N1 viral infections have been reported in most areas of the world and are a major cause of severe acute pneumonia, which often results in acute lung injury (ALI) and acute respiratory distress syndrome (ARDS) with a high risk of morbidity and mortality [2]. Therefore, H1N1 epidemics and pandemics represent a severe challenge and a significant threat to public health.

IAV, like other viruses, requires the host cell machinery to produce infectious progeny. During the early stages of the viral life cycle, viruses enter the cell via receptor-mediated endocytosis and are trafficked to the late endosome, where they fuse and release their genome into the cell. The genome segments are imported into the nucleus and catalysed by the viral polymerase to accomplish transcription and replication. The newly synthesized genome segments are exported from the nucleus and assembled at the cell membrane. In the final step, the budding virus particle is released into the extracellular environment from the host cell membrane [3]. Simultaneously with virus replication, both innate and adaptive immune responses are triggered shortly after infection. Host cells release cytokines as well as chemokines after activation. However, inordinate or unbalanced immune response can result in severe inflammation, including excessive recruitment of neutrophils and mononuclear cells to the lungs, which damages lung tissue, reduces respiratory capacity, and causes severe disease and mortality [4,5]. Therefore, limiting viral replication is the first step towards the treatment of IAV infection.

Autophagy is a complicated and strictly regulated process, involving degradation and turnover of aged proteins and damaged organelles [6]. Many studies have shown that autophagy functions as an adaptive cellular mechanism to deal with stressful stimuli such as starvation, oxidative stress, and pathogen invasion [7,8]. It has been reported recently that IAV NP- and M2-mediated autophagy promotes IAV replication by regulating the AKT-mTOR signalling pathway *in vitro* [9]. During autophagy, microtubule-associated protein 1A/1B-light chain 3 (LC3-I) is lipidated to form LC3-II, which serve as an indicator of autophagic activity and flux. LC3-II directly binds the selective autophagy cargo receptor p62/SQSTM1 (sequestosome-1) and interacts with M2 and NP of IAV to increase viral ribonucleoprotein (vRNP) export and infectious viral particle formation [9,10]. Besides the AKT-mTOR signalling pathway, AMP-activated protein kinase (AMPKα) signalling also initiates autophagy under cellular energy stress. Reduction in cellular ATP levels initiates autophagy through AMPKα, which phosphorylates and activates Unc-51-like autophagy activating kinase 1 (ULK1 kinase) to generate energy by increasing glucose uptake and glycolysis [11,12]. However, the function of AMPKα-ULK1 signalling in IAV infection and replication remains largely unknown.

Host factors are involved in every step of the life cycle of IAV, including cellular proteins and RNAs, which can be simply divided into those that support viral replication and those that play an antiviral role [13]. Hypoxia-inducible factor 1 (HIF-1) is a major transcription factor that allows mammalian cells to adapt to low oxygen tension (hypoxia). HIF-1 is a heterodimeric protein that consists of two proteins, HIF-1α and HIF-1β. HIF-1β is the constitutively expressed subunit, but the expression of HIF-1α is upregulated rapidly in response to hypoxia [14]. Under normoxic conditions, HIF-1α is hydroxylated at conserved proline residues by the prolyl hydroxylases (PHDs), which are oxygen dependent. Hydroxylated HIF-1α undergoes rapid proteasomal degradation by the von Hippel-Lindau (VHL) E3 ubiquitin ligase-mediated ubiquitination. This process results in low basal HIF-1α levels. Hypoxic conditions result in PHD inhibition and lower HIF-1α degradation, which results in HIF-1α accumulation and transcription of HRE (hypoxia response elements)-containing genes [15]. It has been reported that HIF-1 activates the transcription of many genes that are involved in glucose metabolism [16], inflammation [17,18], angiogenesis [19], cell proliferation/survival and invasion/metastasis [20]. However, the role of HIF-1α in IAV infection of alveolar type II epithelial cells (AEC2) is still elusive and deserves further investigation.

In this study, we provide molecular insights into how HIF-1α regulates the replication of IAV by interfering with AMPKα signalling-mediated autophagy in AEC2. We first demonstrated that epithelial cell-specific *Hif1α* knockout mice infected with IAV had more IAV replication in the lung and developed severe lung inflammation. Knockdown of HIF-1α in A549 promoted IAV replication *in vitro* by reducing glycolysis and augmenting AMPKα-ULK1-mediated autophagy. The findings have provided us novel therapeutic targets for dealing with IAV infection.

## Materials and methods

### Cells and influenza A virus

Cell cultures were maintained in a humidified atmosphere at 37°C with 5% CO_2_. A549 (ATCC CCL-185) cells were cultured in F-12 NUTRIENT MIX medium supplemented with 10% fetal bovine serum (FBS), and penicillin/streptomycin. MDCK.2 (ATCC CRL-2936) and 293 T cells were cultured in Dulbecco’s Modified Eagle Medium (DMEM) supplemented with 10% FBS, and penicillin/ streptomycin. A/Puerto Rico/8/1934 H1N1 (A/PR/8) influenza virus was stored in our laboratory. The viruses were grown in the chorioallantoic fluid of 9-day-old specific-pathogen-free (SPF) embryonated chicken eggs (Merial Vital Laboratory Animal Technology Co., Ltd., Beijing, China).

### Mice and virus infection

*Hif1α*^fl/fl^ mice and *Spc-Cre*^+^ mice were generated in a C57BL/6 background. *Hif1α*^fl/fl^ mice were from Jackson Laboratories (stock number: 007561). The sequence of *Hif1α*^fl/fl^ genotyping primer is:

5′-TGCTCATCAGTTGCCACTT-3′(forward) and

5′-GTTGGGGCAGTACTGGAAAG-3′(reverse).

*Spc-Cre*^+^ mice were kindly provided as gift from Prof. Kaifeng Xu (Chinese Academy of Medical Sciences). The surfactant protein C (SPC) is exclusively expressed in the type II alveolar epithelial cells [21]. The sequence of *Spc-Cre*^+^ genotyping primer is:

5′-ATCACTCAGGGCTCTCAGAG-3′(forward) and

5′-CCATTCAGCACACATGAATG-3′(reverse).

These mice were bred and maintained in an SPF animal facility at Institut Pasteur of Shanghai. All the protocols and procedures were conducted in compliance with a protocol approved by the Institutional Animal Care and Use Committee at Institut Pasteur of Shanghai. Mice were anesthetized with 80 mg/Kg pentobarbital sodium dissolved in phosphate-buffered saline (PBS) by intraperitoneal injection. Anesthetized mice were infected intranasally with 20 μl influenza A/PR/8 virus diluted in PBS. Mice in the control group were anaesthetized and inoculated with PBS.

### Antibodies and chemical reagents

AMPKα (5832S), Phospho-ULK1 (Ser555) (5869S), Phospho-ULK1 (Ser757) (14202S), Phospho-Raptor (Ser792) (2083S), ULK1 (D8H5) (8054 T), SQSTM1/p62 (Bimake, Houston, TX, USA), LC3A/B (4108S), Phospho-AMPKα (Thr172) (50081S), RIG-I (3743S), Phospho-Stat1 (Ser727) (8826S), Phospho-Stat1 (Tyr701) (9167S), Stat1 (9172S), Phospho-p38 MAPK (Thr180/Tyr182) (9211S) and Beclin-1 (D40C5) (3495S) antibodies were from Cell Signaling (Danvers, MA, USA). The p38α (sc-535) and NS1 (sc-130568) antibodies were from Santa Cruz Biotechnology (Santa Cruz, CA, USA). Influenza A Virus Nucleoprotein (ab128193), HIF-1-alpha (ab113642) and Influenza A Virus Nucleoprotein (FITC) (ab20921) antibodies were from ABCAM (Cambridge, MA, USA). β-actin (EM32011-02) and GAPDH (EM32010-02) antibodies were from EMAR (EMAR, Beijing, China). Mouse CD326 (Ep-CAM) APC (118213) were from Biolegend (San Diego, CA, USA). Prosurfactant Protein C (proSP-C) (AB3786) antibody was from Merck/Millipore (Merck KGaA, Darmstadt, Germany). Human/Mouse HIF-1 alpha (APC) antibody was from R&D (R&D Systems, Minneapolis, USA). Influenza A Virus PB2 antibody (PA532220) was from Pierce (Dallas, TX, USA). IFITM3 Antibody (11714-1-AP) was from Proteintech (Rosemont, USA). Chloroquine diphosphate salt solid (C6628-25G) was from Sigma/flu/Ald (Merck KGaA, Darmstadt, Germany). Rapamycin (S1039), LY294002 (S1105), and 3-MA (S2767) were from Selleck Chemicals (Selleck, Houston, TX, USA).

### Virus titration by foci assay

MDCK (1 × 10^5^/well) cells were seeded in 96-well culture plates and incubated at 37°C in 5% CO_2_ for 24 h. For foci assay, cell culture supernatant or lung homogenates were harvested. Serial 10-fold dilutions of lung samples in DMEM containing 0.1% BSA were prepared and added to MDCK cells. The plates were incubated at 37°C in 5% CO_2_ for 1 h. The media was then aspirated and replaced with 100 µl of 1% Avicell overlay and incubated at 37°C for 24 h. Subsequently, the plates were fixed with 4% formalin in PBS for 10 min at room temperature (RT). Then the cells were washed and incubated for 10 min with 100 µl/well of Quencher (0.5% Triton × 100, 20 mM glycine in PBS). After 10 min the cells were washed with Wash Buffer (0.5% Tween 20 in PBS) and blocked with 50 µl Blocking Buffer (0.5% Tween 20, 1% BSA in PBS) at 37°C in 5% CO_2_ for 30 min. The primary antibody (anti-influenza Nucleocapsid NP polyclonal goat antibody from Virostat, Portland, USA) and the secondary antibody (anti-goat-HRP from KPL, Gaithersburg MD, USA) were diluted 1∶1000 in Blocking Buffer. 50 µl of the primary antibody was added to each well and incubated at RT for 1 h. The cells were then incubated with 50 µl of the secondary antibody for 45 min and then incubated with 50 µl of the substrate (True Blue from KPL) until blue spots from infected cell foci appeared. Foci were counted and viral titres calculated as focus forming units (FFU/lung).

### RNA, cDNA, and RT-qPCR

Total RNA was extracted from homogenized lungs or cultured cells using TRIzol reagent (Invitrogen) based on the manufacturer’s instructions. RNA was quantified and cDNA was synthesized using a reverse transcriptase kit (Tiangen, Beijing, China), followed by quantitative real-time polymerase chain reaction (RT-qPCR) analysis (SYBR Green, Selleck).

The sequences of the primers used are as follows:

PR8 *M gene* 5′-AAGACCAATCCTGTCACCTCTGA-3′(forward) and

    5′-CAAAGCGTCTACGCTGCAGTCC-3′(reverse);

Murine primers for animal experiments:

*Gapdh* 5′-AGGTCGGTGTGAACGGATTTG-3′(forward) and

    5′-TGTAGACCATGTAGTTGAGGTCA-3′(reverse);

*Hif1α* 5′-ACTCATCCATGTGACCATGAG-3′(forward) and

    5′-TGACTTGATGTTCATCGTCCTC-3′(reverse);

*Cxcl2* 5′-CGCTGTCAATGCCTGAAG-3′(forward) and

    5′-GGCGTCACACTCAAGCTCT-3′(reverse);

*Cxcl10* 5′-CCGGAATCTAAGACCATCAAG-3′(forward) and

    5′-GAGGCTCTCTGCTGTCCATC-3′(reverse);

*Mcp1* 5′-GAAGGAATGGGTCCAGACAT-3′(forward) and

    5′-ACGGGTCAACTTCACATTCA-3′(reverse);

*Ifnβ* 5′-AGATCAACCTCACCTACAGG-3′(forward) and

    5′-TCAGAAACACTGTCTGCTGG-3′(reverse);

Homo sapiens primers for cell line:

*HIF1A* 5′-TGCTCATCAGTTGCCACTTC-3′(forward) and

    5′-TGCTCATCAGTTGCCACTTC-3′(reverse);

*ACTB* 5′-CTCTTCCAGCCTTCCTTCCT-3′(forward) and

    5′-AGCACTGTGTTGGCGTACAG-3′(reverse);

*IFNB* 5′-ATGACCAACAAGTGTCTCCTCC-3′(forward) and

    5′-GGAATCCAAGCAAGTTGTAGCTC-3′(reverse);

*CXCL2* 5′-TGCAGGGAATTCACCTCAAG-3′(forward) and

    5′-TGAGACAAGCTTTCTGCCCA-3′(reverse);

*MCP1* 5′-CAGCCAGATGCAATCAATGCC-3′(forward) and

    5′-TGGAATCCTGAACCCACTTCT-3′(reverse);

*IL6* 5′-AGAGGCACTGGCAGAAAACAAC-3′(forward) and

    5′-AGGCAAGTCTCCTCATTGAATCC-3′(reverse);

*CXCL11* 5′-GACGCTGTCTTTGCATAGGC-3′(forward) and

    5′-GGATTTAGGCATCGTTGTCCTTT-3′(reverse);

*CCL20* 5′-TGCTGTACCAAGAGTTTGCTC-3′(forward) and

    5′-CGCACACAGACAACTTTTTCTTT-3′(reverse);

*CXCL8* 5′-TTTTGCCAAGGAGTGCTAAAGA-3′(forward) and

    5′-AACCCTCTGCACCCAGTTTTC-3′(reverse);

*CCL5* 5′-CCAGCAGTCGTCTTTGTCAC-3′(forward) and

    5′-CTCTGGGTTGGCACACACTT-3′(reverse);

*LDHA* 5′-ATGGCAACTCTAAAGGATCAGC-3′(forward) and

    5′-CCAACCCCAACAACTGTAATCT-3′(reverse);

*HK2* 5′-GAGCCACCACTCACCCTACT-3′(forward) and

    5′-GAGCCACCACTCACCCTACT-3′(reverse);

*GLUT1* 5′-GGCCAAGAGTGTGCTAAAGAA-3′(forward) and

    5′-ACAGCGTTGATGCCAGACAG-3′(reverse);

*PKM2* 5′-ATGTCGAAGCCCCATAGTGAA-3′(forward) and

    5′-TGGGTGGTGAATCAATGTCCA-3′(reverse);

*GAPDH* 5′-GCCCCACTTGATTTTGGAGG-3′(forward) and

    5′-GCAAATTTCCATGGCACCGT-3′(reverse).

## mRNA library construction and RNA-Seq

Total RNA was extracted from A549 cells mock-infected or infected with PR8 at MOI of 2 at 24 hpi using TRIzol reagent (Invitrogen) based on the manufacturer’s instructions. The RNA was treated with DNase I for 30 min at 37°C to remove residual DNA. mRNA was purified with oligo (dT) beads. Then, the purified mRNA was fragmented into small pieces with fragment buffer at appropriate temperature. The first-strand and second-strand cDNA was generated. Afterwards, A-Tailing Mix and RNA Index Adapters were added by incubating to end repair. The cDNA fragments were further amplified by PCR, and the products were purified by Ampure XP Beads, then dissolved in EB solution. The product was validated on the Agilent Technologies 2100 bioanalyzer for quality control. The amplified PCR products from previous step were denatured and circularized by the splint oligo sequence to get the library. The single strand circle DNA (ssCir DNA) was formatted as the final library. The final library was amplified to make DNA nanoball (DNB) which had more than 300 copies of one molecular, DNBs were loaded into the patterned nanoarray and single end 50 bases reads were generated on BGIseq500 platform (BGI-Shenzhen, China). The gene expression levels were calculated in fragments per kilobase transcriptome per million mapped reads (FPKM).

### Western blotting analysis

Denatured proteins were equally loaded on SDS–PAGE gel. The proteins were resolved by electrophoresis, transferred to PVDF membranes, hybridized with indicated primary antibodies and corresponding HRP labelled secondary antibodies, and visualized using a Western ECL Substrate (Tanon, Shanghai, China). The bands were analysed and quantitated using Image J software.

### Immunofluorescent microscopy of autophagy

For the detection of autophagosomes, cells were infected with lentiviruses expressing GFP-LC3B. After 48 h of lentiviral infection, these cells were used for next experiments. Following corresponding experimental treatment, cells were fixed in 4% formaldehyde and permeabilized in 0.5% Triton-X 100. Cell nuclei were stained with DAPI dye and slides were imaged on a laser-scanning confocal microscope (Olympus FV-1200). Images were quantified by Image J Pro.

### Short hairpin RNA (shRNA)–mediated RNA interference

Short hairpin RNA (shRNA) sequences, synthesized by Nanjing Genewis Biotechnology, were inserted into pLKO.1 plasmid between the EcoRI and NheI restriction sites. The shRNA PLKO.1 construct was introduced into target cells via lentiviral transduction. The knockdown assay primer sequences were, Scramble CAACAAGATGAAGAGCACCAA; HIF-1α CCGCTGGAGACACAATCATAT; BECN1 CTCAGGAGAGGAGCCATTTAT; p62 GAGGATCCGAGTGTGAATTTC; AMPKα GAAGGTTGTAAACCCATATTA; ULK1 GCCCTTTGCGTTATATTGTAT.

### Histology

After mice were anesthetized and sacrificed with 80 mg/Kg pentobarbital sodium, the chest and abdomen were rapidly opened. The lungs were removed and fixed in 4% paraformaldehyde overnight at RT. The tissues were embedded in paraffin, 5 μm sections were cut and stained with haematoxylin and eosin (H&E).

### Isolation of mouse lung primary cells

The chest cavity of the mice was opened and lung vascular beds flushed by injecting 5 ml of chilled (4°C) PBS into the right ventricle. Lungs were excised, minced and digested in DMEM/ F-12 K (Gibco) collagenase (Sigma) solution at 37°C for 1 h. Dissociated cells were transferred to a 15 ml conical tube and centrifuged for 10 min (335 × g, 4°C). The red blood cells were lysed by the addition of 3 ml ACK lysing buffer for 5 min at RT. The cells were washed with 12 ml cold PBS/0.5% BSA for 10 min (335 × g, 4°C). The cell pellets were resuspended in 5 ml of cold PBS/0.5% BSA and filtered through a 70 μm nylon mesh.

### Flow cytometry

Cells isolated from tissues or cultured cells were kept at 4°C, and nonspecific binding was blocked with anti-CD16/32 antibody before cell surfaces staining. Surface markers were stained for 20 min on ice in PBS supplemented with 2% FBS. Intracellular markers were stained with Fixation/Permeabilization Solution Kit (BD Biosciences). Dead cells were discarded during analysis by staining with DAPI. All the antibodies used are summarized in **Antibodies and Chemical reagents**. Flow cytometry analysis was performed on LSRII or Fortessa (BD Biosciences), and flow sorting was performed on FACSAriaII (BD Biosciences). Data was analysed using the FlowJo software (Tree Star, Ashland, OR).

### Statistical analysis

Statistical analysis was performed using the GraphPad Prism software (GraphPad, San Diego, CA). Two-tailed Students’ *t*-test was used to determine significance. The Gehan-Breslow-Wilcoxon Test was used to compare survival data. The results are shown as mean ± SD.

## Results

### Deficiency of HIF-1α in AEC2 aggravates weight-loss and reduces survival after IAV infection

**Figure 1.**
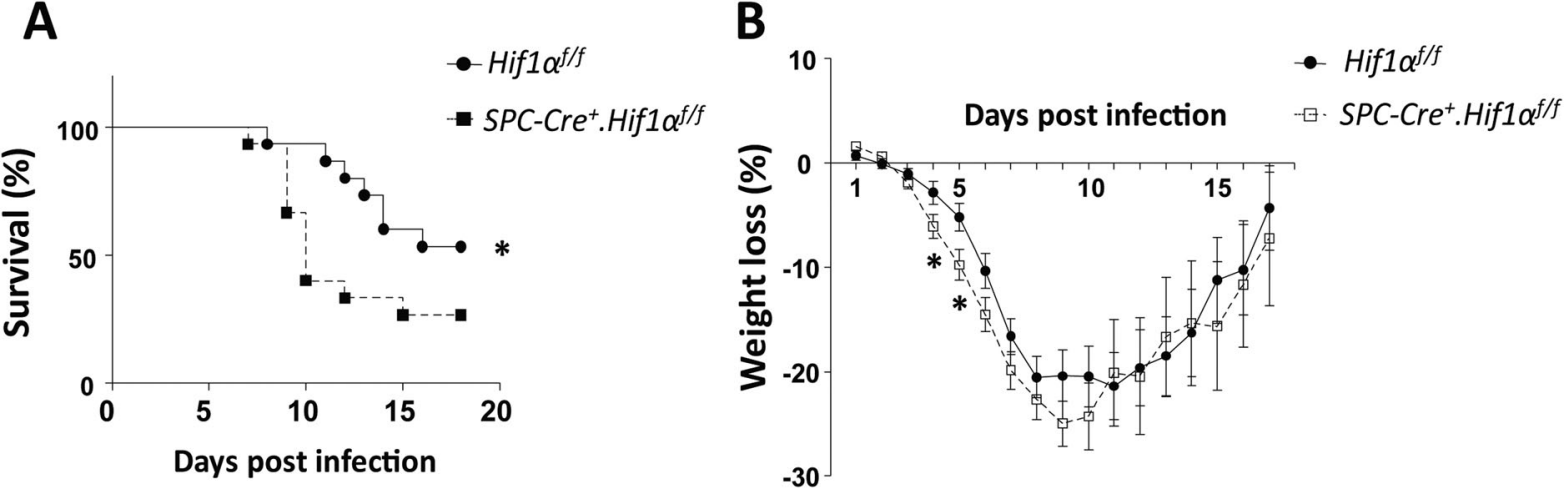
HIF-1α deletion in AEC2 leads to increased mortality and weight loss after IAV infection. Survival rate (A) and weight loss (B) of eight to ten weeks old male *Hif1α*^fl/fl^ (*n* = 15) and *Spc-Cre*^+^.*Hif1α*^fl/fl^ (*n* = 15) mice infected with 5 × 10^3^ FFU mouse-adapted PR8. The data of survival rate was analysed by Gehan-Breslow-Wilcoxon Test. The data of weight loss was analysed by student’s *t*-test (two-tailed) and presented as mean ± SD (*p < 0.05, **p < 0.01).

To assess the *in vivo* role of HIF-1α in AEC2 during influenza virus infection, we first generated *Spc-Cre*^+^.*Hif1α*^fl/fl^ mice and then established an IAV infection mouse model. Upon intranasal infection of mice with a low lethal dose of 3.5 LD50 mouse-adapted PR8 (A/PuertoRico/8/34 H1N1), *Spc-Cre^+^.Hif1α*^fl/fl^ mice showed increased mortality (Figure 1(A)) and weight loss (Figure 1(B)) compared to the PR8-infected control mice.

### Deficiency of HIF-1α in AEC2 worsens lung inflammation and injury after IAV infection

**Figure 2.**
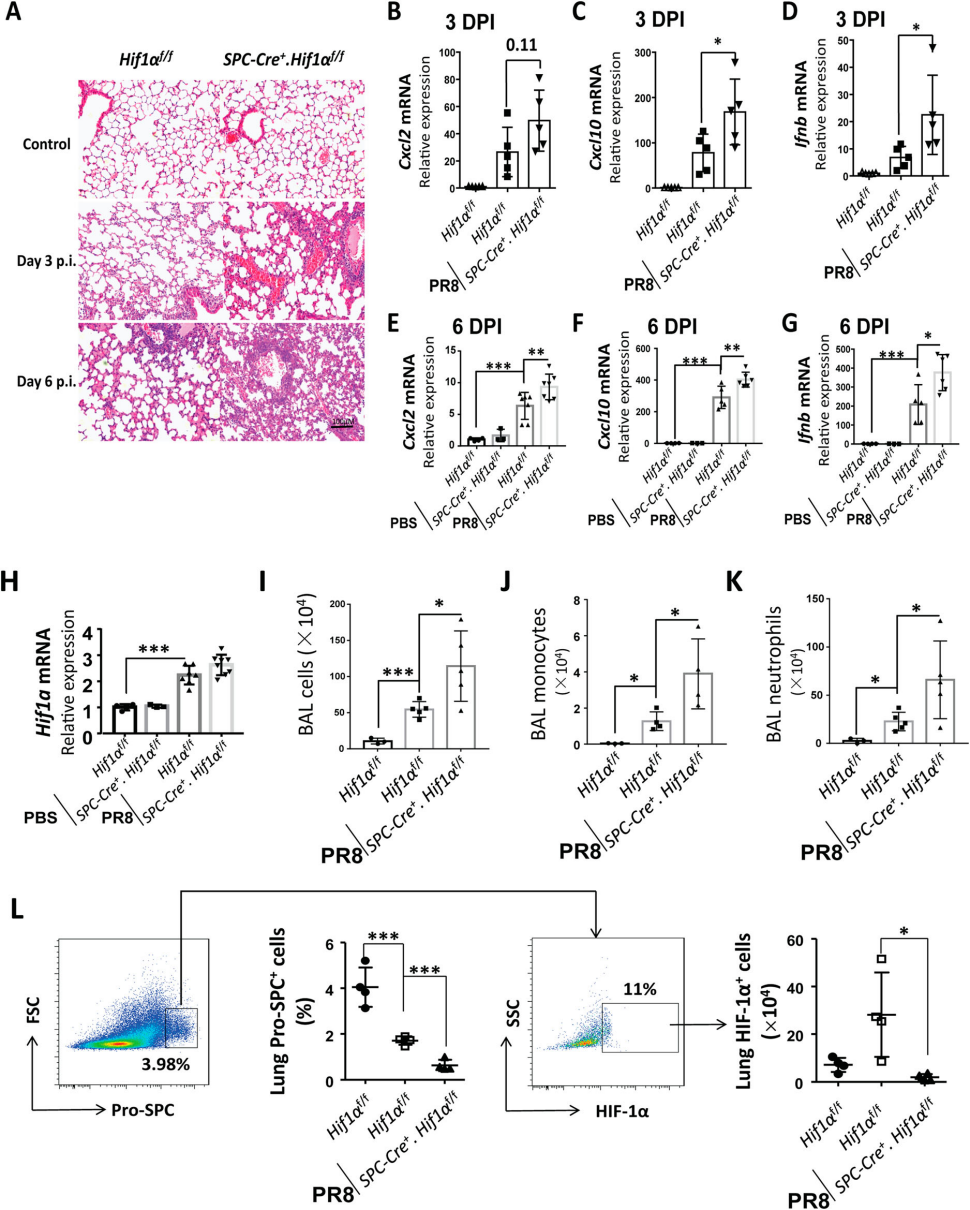
HIF-1α deletion in AEC2 increases influenza-induced lung inflammation and injury. (A) *Hif1α*^fl/fl^ and *Spc-Cre*^+^.*Hif1α*^fl/fl^ mice were infected with 5 × 10^3^ FFU mouse-adapted PR8. Serial lung sections of representative lungs at day 3 and 6 post IAV infection were stained with haematoxylin and eosin. (B-D) Quantitative mRNA expression of inflammatory genes (*Cxcl2, Cxcl10, Ifnb*) in the lung of *Hif1α*^fl/fl^ and *Spc-Cre*^+^.*Hif1α*^fl/fl^ mice at day 3 post IAV infection with 5 × 10^3^ FFU mouse-adapted PR8. (E-G) Quantitative mRNA expression of inflammatory genes (*Cxcl2, Cxcl10, Ifnb*) in the lung of *Hif1α*^fl/fl^ and *Spc-Cre*^+^.*Hif1α*^fl/fl^ mice at day 6 post IAV infection with 5 × 10^3^ FFU mouse-adapted PR8. (H) Quantitative mRNA expression of *Hif1α* in the lung of *Hif1α*^fl/fl^ and *Spc-Cre*^+^.*Hif1α*^fl/fl^ mice at day 6 post IAV infection with 5 × 10^3^ FFU mouse-adapted PR8. (I-K) Number and flowcytometry analysis of BAL cells isolated from *Hif1α*^fl/fl^ and *Spc-Cre*^+^.*Hif1α*^fl/fl^ mice at day 6 post IAV infection with 5 × 10^3^ FFU mouse-adapted PR8. Monocytes and neutrophils were labelled by surface marker Ly6C and Ly6G. (L) Flow cytometry analysis of pro-SPC^+^ and HIF-1α^+^ cells in digested lung cells from *Hif1α*^fl/fl^ and *Spc-Cre*^+^.*Hif1α*^fl/fl^ mice at day 6 post IAV infection with 5 × 10^3^ FFU mouse-adapted PR8. N=3-8 in each group. The data was analysed by Student’s *t*-test (two-tailed) and is presented as mean ± SD (*p < 0.05, **p < 0.01, ***p < 0.001).

Histological analyses of infected lungs revealed that more alveolar regions were injured in *Spc-Cre*^+^.*Hif1α*^fl/fl^ mice compared to control *Hif1α*^fl/fl^ mice at day 3 and 6 post infection. Furthermore, infected *Spc-Cre*^+^.*Hif1α*^fl/fl^ mice showed a higher number of lung infiltrating cells compared to *Hif1α*^fl/fl^ mice (Figure 2(A)). Using the same infection conditions, we sacrificed mice 3 and 6 days after IAV infection and analysed the total lung mRNA levels. We measured the expression of inflammation-related genes, *Cxcl2* (a chemokine for neutrophils) (Figure 2(B,E)), *cxcl10* (Figure 2(C,F)), and *Ifn-β* (Type I interferon induced by virus infection) (Figure 2(D,G)) by RT-qPCR. Data showed that mRNA levels of lung *Cxcl2*, *cxcl10* and *Ifn-β* were increased in IAV-infected wild-type mice compared to PBS-treated wild-type mice and expression was even higher in *Spc-Cre*^+^.*Hif1α*^fl/fl^ mice. To determine the level of HIF-1α in the infected lung, we further analysed the whole lung HIF-1α mRNA levels by RT-qPCR. We found that HIF-1α mRNA levels in the lung were significantly increased after IAV infection (Figure 2(H)). The total bronchoalveolar lavage (BAL) cell number was significantly higher in *Spc-Cre*^+^.*Hif1α*^fl/fl^ mice compared to control *Hif1α^f^*^l/fl^ mice during IAV infection (Figure 2(I)). We further used flow cytometry to analyse monocytes and neutrophils labelled with Ly6C and Ly6G in BAL cells. Data showed that Ly6G^-^Ly6C^+^ monocytes (Figure 2(J)) and Ly6G^+^Ly6C^mid^ neutrophils (Figure 2(K)) in BAL was also significantly increased in *Spc-Cre*^+^.*Hif1α*^fl/fl^ mice compared to control *Hif1α*^fl/fl^ mice.

Since HIF-1α is specifically knocked out in AEC2 in *Spc-Cre*^+^.*Hif1α*^fl/fl^ mice, we further measured the levels of HIF-1α in AEC2 in the digested lung cells by flow cytometry analysis**.** The surfactant protein C (SPC) is exclusively expressed in the type II alveolar epithelial cells [21]. The results indicated that HIF-1α expression in SPC^+^ cells were significantly increased after IAV infection in control mice and reduced in *Spc-Cre*^+^.*Hif1α*^fl/fl^ mice (Figure 2(L)). A previous study has reported that IAV infection-induced lung injury could be reflected by decreased lung SPC^+^ cells [22]. In corroboration, we found that lung SPC^+^ cells were reduced after IAV infection and further reduced in *Spc-Cre*^+^.*Hif1α*^fl/fl^ mice (Figure 2(L)). These findings suggest that deficiency of HIF-1α in AEC2 contributes to increased lung inflammation and injury during IAV infection.

### Deficiency of HIF-1α in AEC2 promotes IAV replication in the lung

**Figure 3.**
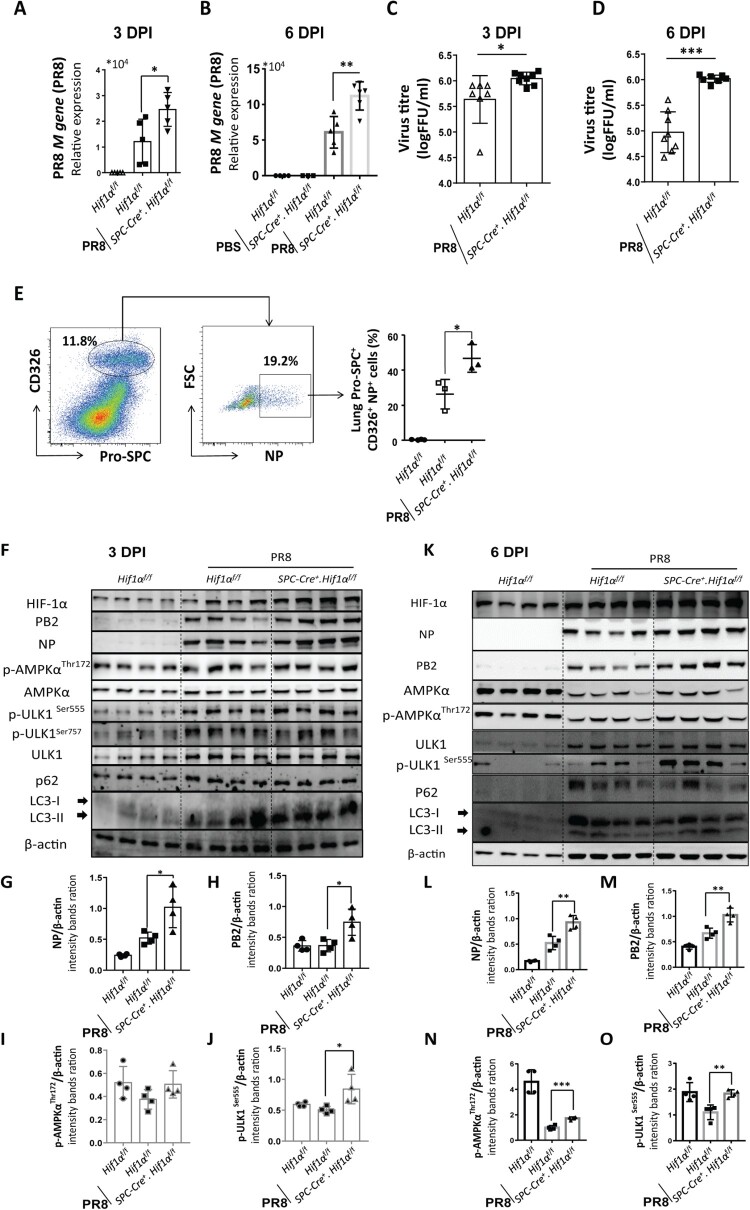
HIF-1α deletion in AEC2 promotes IAV replication in the lung. (A-B) Quantitative mRNA expression of *PR8 M* gene from the lung of *Hif1α*^fl/fl^ and *Spc-Cre*^+^.*Hif1α*^fl/fl^ mice at day 3 (A) and 6 (B) post IAV infection with 5 × 10^3^ FFU mouse-adapted PR8. (C-D) Virus titres of the lung of *Hif1α*^fl/fl^ and *Spc-Cre*^+^.*Hif1α*^fl/fl^ mice at day 3 (C) and 6 (D) post IAV infection with 5 × 10^3^ FFU mouse-adapted PR8. (E) Flow cytometry analysis of NP^+^ AEC2 cells in digested lung cells of *Hif1α*^fl/fl^ and *Spc-Cre*^+^.*Hif1α*^fl/fl^ mice at day 6 post IAV infection with 5 × 10^3^ FFU mouse-adapted PR8. The AEC2s were labelled with fluorescent anti-pro-SPC and CD326 antibodies. (F) Immunoblot analysis of the supernatant of lung homogenates from *Hif1α*^fl/fl^ and *Spc-Cre*^+^.*Hif1α*^fl/fl^ mice at day 3 post IAV infection with 5 × 10^3^ FFU mouse-adapted PR8(G-J). Intensity analysis of proteins in (F) was performed. (K) Immunoblot analysis of the supernatant of lung homogenates from *Hif1α*^fl/fl^ and *Spc-Cre*^+^.*Hif1α*^fl/fl^ mice at day 6 post IAV infection with 5 × 10^3^ FFU mouse-adapted PR8. (L-O) Intensity analysis of proteins in (K) was performed. β-actin was used as a loading control. The intensities of the protein bands were quantified using Image J software. The data was analysed by Student’s *t*-test (two-tailed) were presented as mean ± SD (*p < 0.05, **p < 0.01, ***p < 0.001).

To address why HIF-1α deletion in AEC2 led to aggravated lung inflammation and injury, we first evaluated IAV replication in the lung. RT-qPCR results showed that *M gene* levels of IAV were significantly higher in *Spc-Cre*^+^.*Hif1α*^fl/fl^ mice compared to *Hif1α*^fl/fl^ mice at 3 (Figure 3(A)) and 6 (Figure 3(B)) days after IAV infection. We further measured virus titres in the supernatant of lung homogenates from *Hif1α*^fl/fl^ and *Spc-Cre*^+^.*Hif1α*^fl/fl^ mice at day 3 and 6 post IAV infection with 5 × 10^3^ FFU mouse-adapted PR8. Results showed that virus titres were significantly increased in *Spc-Cre*^+^.*Hif1α*^fl/fl^ mice compared to *Hif1α*^fl/fl^ mice at 3 (Figure 3(C)) and 6 (Figure 3(D)) days after IAV infection. CD326 (epithelial cell adhesion molecule, EpCAM) is a marker of epithelial cells. CD326 and SPC were used as markers of AEC2. Using the same flow cytometry procedure in Figure 2(L), we found that IAV infected AEC2 were higher in *Spc-Cre*^+^.*Hif1α*^fl/fl^ mice compared to *Hif1α*^fl/fl^ mice (Figure 3(E)). Western blot results of whole lung homogenates also showed that HIF-1α deletion in AEC2 led to enhanced IAV replication (Figure 3(F,K)). Together, these data suggest that deficiency of HIF-1α in AEC2 promotes IAV replication in the lung *in vivo*.

We next investigated the signalling response caused by HIF-1α deletion in AEC2 during IAV infection. We found that AMPKα-ULK1 signalling was more active in Spc-Cre^+^.*Hif1α*^fl/fl^ mice compared to *Hif1α*^fl/fl^ mice (Figure 3(F,K)). AMPKα-ULK1 signalling can initiate autophagy under cellular energy and is tightly correlated with HIF-1α-mediated metabolism. Moreover, autophagy is essential for IAV replication [9]. Our results showed that the ratio of lipidated LC3-II and LC3-I, which increase with the autophagy, was seemed to be higher in Spc-Cre^+^.*Hif1α*^fl/fl^ mice compared to *Hif1α*^fl/fl^ mice after IAV infection. These findings hint us that AMPKα-ULK1 signalling-mediated autophagy may play an important role in IAV replication. Thus, we next explored the signalling mechanism underlying this phenotype wherein a deficiency of HIF-1α enhanced IAV replication in AEC2.

### The mRNA and protein levels of HIF-1α are upregulated after IAV infection *in vitro*

**Figure 4.**
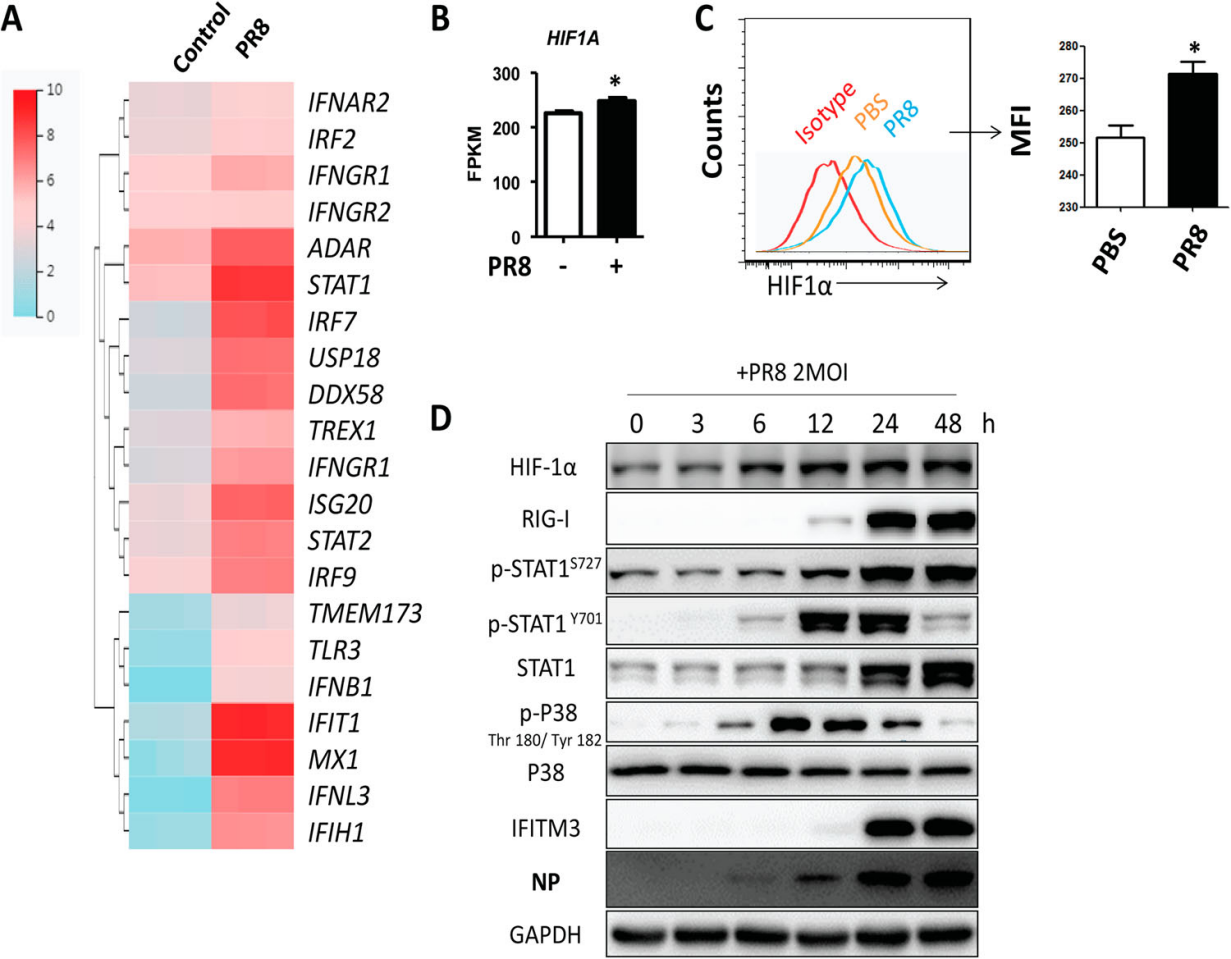
The mRNA and protein levels of HIF-1α are upregulated after IAV infection in *vitro.* (A) Heatmap of the innate immune response related-genes in A549 cells mock-infected or infected with 2 MOI PR8 virus by FPKM value of RNAseq at 24 h post infection (hpi). (B) FPKM value analysis of *HIF1A* gene expression in A549 cells mock-infected or infected with 2 MOI PR8 virus at 24 hpi. (C) A549 cells were mock-infected or infected with the PR8 virus at MOI of 2. Cells were collected at 24 hpi and were subjected to flow cytometry analysis. Mean fluorescence intensity (MFI) of HIF-1α expression analysis showed. (D) A549 cells were infected with the PR8 virus at MOI of 2. Cell lysates were collected at 0, 3, 6, 12, 24, and 48 hpi and subjected to Western blot analysis. The data was analysed by Student’s *t*-test (two-tailed) and is presented as mean ± SD (*p < 0.05).

To investigate the effects of IAV infection on HIF-1α expression, we first infected A549 cells with PR8 and then quantified gene expression by RNAseq. First, we analysed the expression of innate immune response-related genes. Our results showed that the interferon stimulated genes (ISGs) were significantly upregulated after PR8 infection (Figure 4(A)). We next analysed HIF-1α gene expression. HIF-1α showed a modest but significant upregulation in A549 cells after IAV infection (Figure 4(B)). Flow cytometry analysis of A549 cells labelled with HIF-1α antibody showed that IAV infection could upregulate HIF-1α level in A549 cells compared to the uninfected controls (Figure 4(C)). In agreement, the protein levels of HIF-1α were also higher in A549 at different time points post-IAV infection (Figure 4(D)). Therefore, our data indicated that IAV infection upregulated HIF-1α mRNA and protein levels in A549 cells.

### HIF-1α knockdown promotes IAV replication accompanied by enhanced autophagy *in vitro*

**Figure 5.**
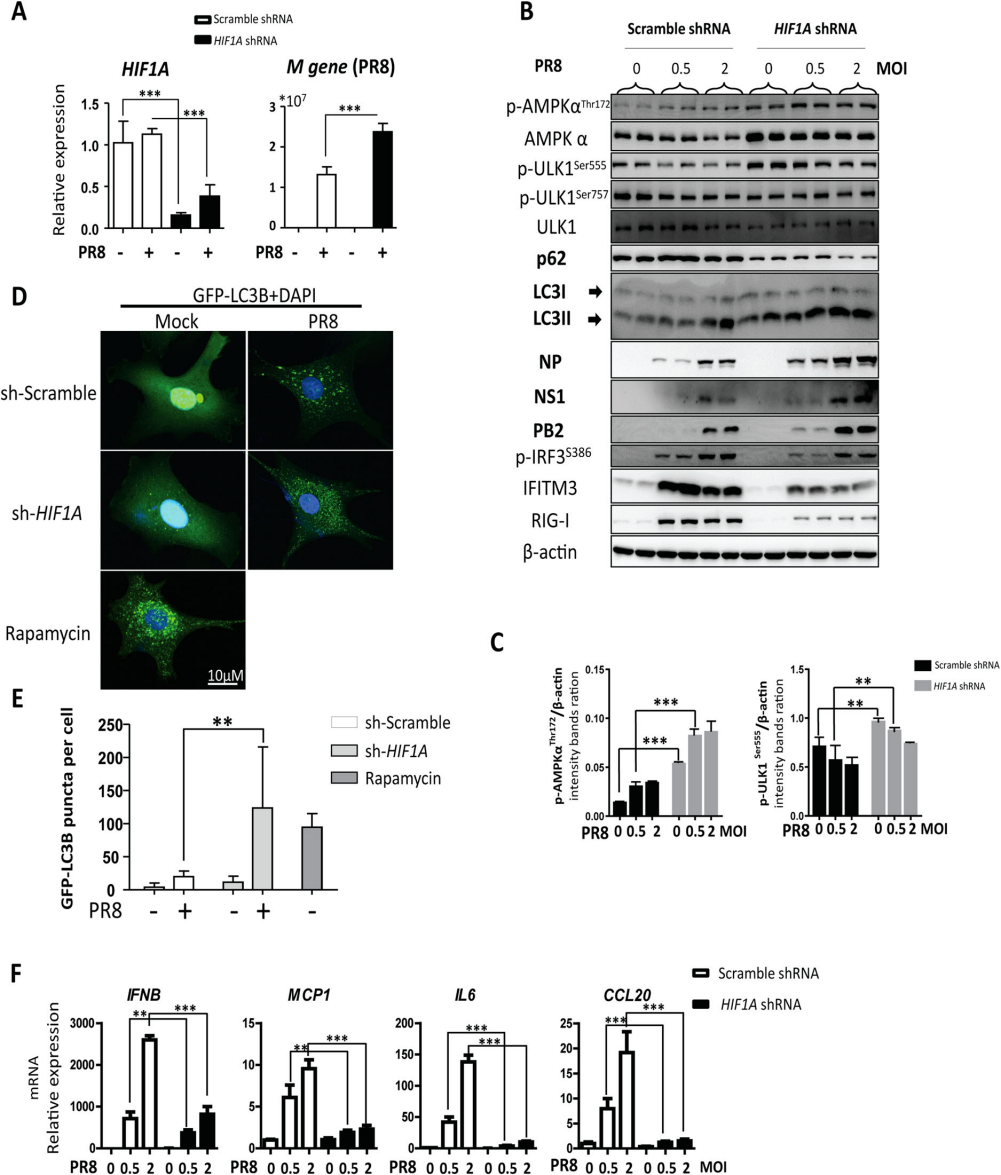
HIF-1α knockdown enhances IAV replication through autophagy. (A) Effect of HIF-1α knockdown on IAV replication in A549 cells. A549 were infected with pLKO.1 scrambled or shRNA-*HIF1A* lentivirus, infected with PR8 at MOI of 2 and harvested at 24 hpi to analyse M gene and *HIF1A* expression by RT-qPCR. (B) Western blot analysis of samples treated and harvested as in (A). (C) Intensity analysis of proteins, p-AMPKα^Thr172^ and p-ULK1^Ser555^, in (B) was performed. (D) A549 cells were infected with GFP-LC3B reporter lentivirus and then transfected with pLKO.1 scrambled or shRNA-HIF1A lentivirus and treated with rapamycin. Representative puncta images of A549-GFP-LC3B cells mock-infected or infected with PR8 at MOI of 2 at 24 hpi. (E) Quantification of GFP-LC3B puncta in (D). (F) Effect of HIF-1α knockdown on transcription of inflammatory cytokines genes (*IFNB*, *MCP1*, *IL6*, *CCL20*) of A549 cells mock-infected or infected with 2 MOI PR8 at 24 hpi. The data was analysed by Student’s *t*-test (two-tailed) and is presented as mean ± SD (*p < 0.05, **p < 0.01, ***p < 0.001).

To further explore the role of HIF-1α on IAV replication, we silenced HIF-1α expression in A549 cells by lentivirus-mediated specific shRNA gene silencing. We found that knockdown of HIF-1α in A549 cells increased IAV replication markedly at the gene expression and protein level compared to the scrambled group (Figure 5(A,B)). It is speculated that HIF-1α may regulate autophagy to facilitate IAV replication. Consistent with the *in vivo* data, we found that LC3-II was increased and p62 was decreased in HIF-1α knockdown cells during IAV infection (Figure 5(B)). We also found that AMPKα-ULK1 signalling was increased in HIF-1α knockdown cells during IAV infection compared to the scrambled group (Figure 5(C)). These results suggested that autophagy in A549 cells was significantly enhanced in the HIF-1α silenced cells. To determine whether the autophagic response is also regulated by HIF-1α during IAV infection in A549 cells, a GFP-LC3B reporter lentivirus was constructed. This tag reporter lentivirus enables the detection of autophagic flux by counting GFP-LC3B puncta. Compared to uninfected cells, the number of fluorescent LC3 puncta noticeably increased in IAV-infected cells. Consistent with the Western blot results shown in Figure 5(B), loss of HIF-1α also markedly increased GFP-LC3B puncta formation (Figure 5(D,E)). Taken together, these results indicate that HIF-1α plays an important role in triggering autophagy during IAV infection *in vitro*.

### HIF-1α is critical for expression of proinflammatory cytokines during IAV infection

It has recently been demonstrated that HIF-1α plays an important role in regulating the expression levels of cytokines such as TNF-α and IL-6. HIF-1α nuclear accumulation led to enhanced proinflammatory cytokine production in IAV-infected A549 cells [23]. To substantiate the role of HIF-1α in IAV induced proinflammatory cytokine production, we analysed the expression levels of some pro-inflammatory cytokines by RT-qPCR. Consistently, knockdown of HIF-1α in A549 cells markedly decreased gene expression level of proinflammatory cytokines (Figure 5(F)). These results suggest that HIF-1α is critical for the expression proinflammatory cytokines during IAV infection.

### Autophagy is vital for IAV replication *in vitro*

**Figure 6.**
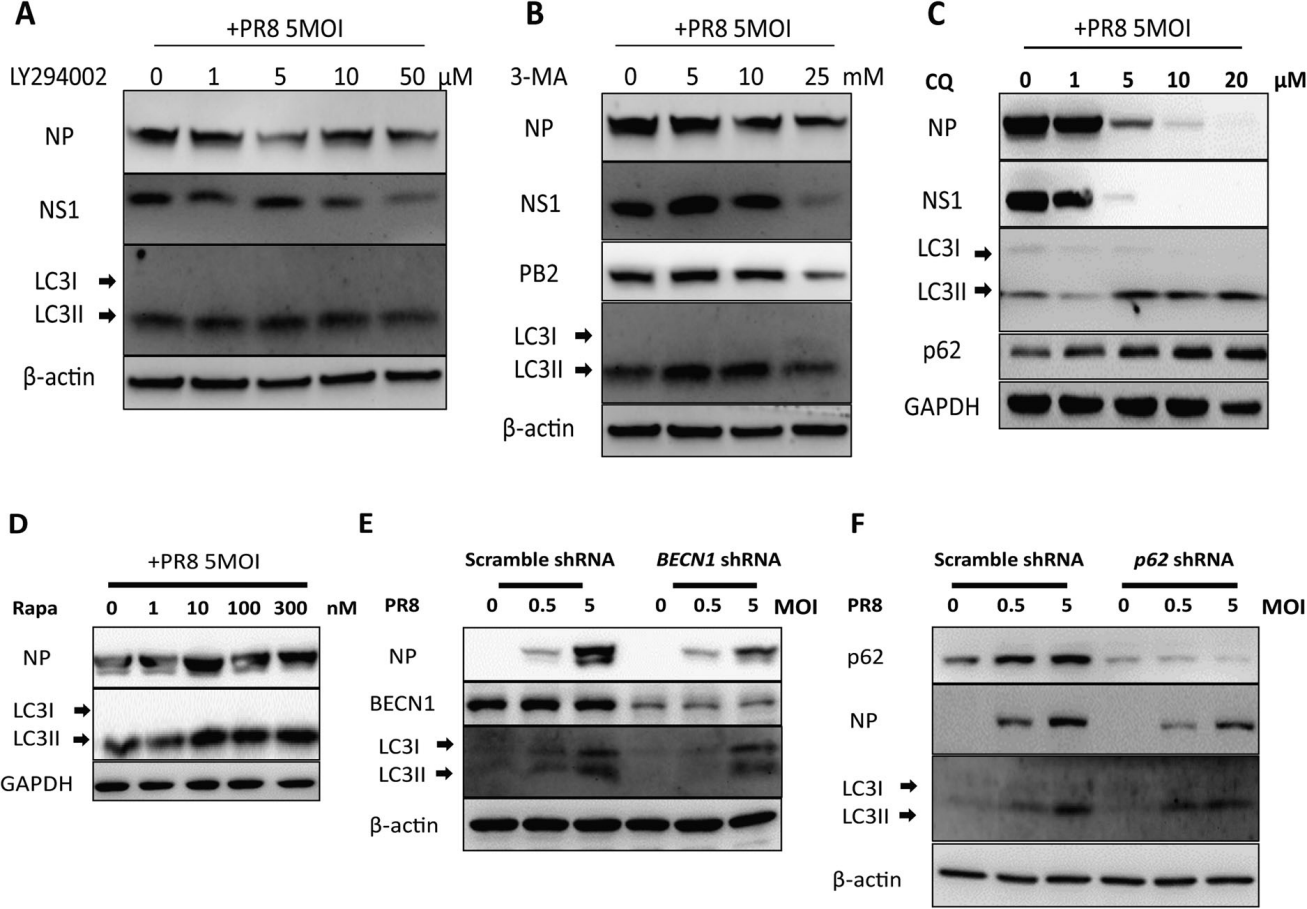
Autophagy inhibition reduces replication of Influenza A Virus *in vitro*. (A-D) A549 cells were pretreated with an increasing dose of LY294002 (A) or 3-MA (B) or chloroquine (C) or Rapamycin (D) for 30 min and then mock-infected or infected with PR8 at MOI of 5. Cell lysates were collected at 24 hpi and were subjected to Western blot analysis. (E-F) Effect of BECN1 and p62 knockdown on IAV replication in A549 cells. A549 cells were infected with pLKO.1 scrambled or shRNA-*BECN1* (E) or shRNA-*p62* (F) lentiviruses, infected with PR8 and harvested at 24 hpi for Western blot analysis. Results are representative of three independent experiments.

To test whether autophagy promotes IAV replication, autophagy was inhibited by applying autophagy inhibitors, LY294002 (Figure 6(A)), 3-MA (Figure 6(B)) and chloroquine (CQ) (Figure 6(C)). As previously reported, our results also showed that IAV propagation was efficiently inhibited after the disruption of autophagy. We further took advantage of rapamycin to induce autophagy and observe the change in viral replication. Our results showed that rapamycin treatment increased the expression of LC3-II and IAV replication compared to untreated cells (Figure 6(D)). Subsequently, the phenomenon was further validated by knocking down the cargo receptor p62/SQSTM1 and BECN1, which not only participates in autophagosome formation but also interacts with different protein complexes regulating the autophagosome-lysosome fusion. In agreement with our inhibitor experiments, knockdown of BECN1 and p62 in A549 cells inhibited IAV replication (Figure 6(E,F)). These results indicated that autophagy regulates IAV replication *in vitro*.

### HIF-1α knockdown inhibits glycolysis and activates AMPKα-ULK1 signalling during IAV infection *in vitro*

**Figure 7.**
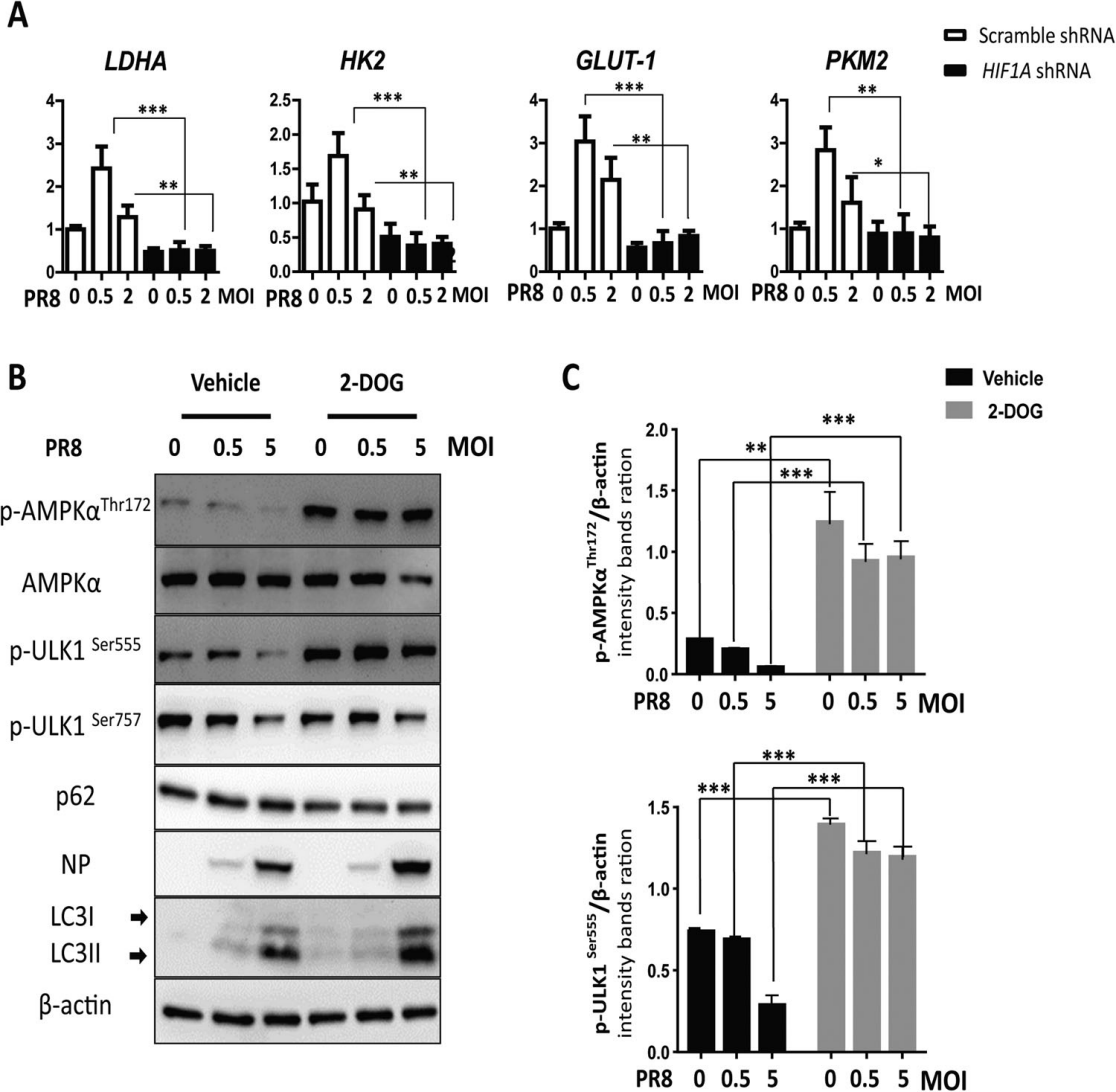
HIF-1α knockdown downregulates glycolysis and activates the AMPKα-ULK1 signaling pathway during IAV infection. (A) Effect of HIF-1α knockdown on the expression of glycolysis genes (*LDHA*, *HK2*, *GLUT-1*, *PKM2*) during IAV infection in A549 cells mock-infected or infected with PR8 at 24 hpi by RT-qPCR. (B) A549 cells were pretreated with 2-DOG or vehicle for 30 min and then were mock-infected or infected with the PR8 virus for 24 h. Cells were harvested and analysed by Western blotting. (C) Intensity analysis of proteins, p-AMPKα^Thr172^ and p-ULK1^Ser555^, in (B) was performed. The data were analysed by Student’s *t*-test (two-tailed) and is presented as mean ± SD (*p < 0.05, **p < 0.01, ***p < 0.001).

Given that HIF-1α-driven glycolysis is one of the most important functions of HIF-1α in metabolism, we measured the expression of several genes involved in the glycolytic pathway. Our results showed that IAV infection upregulated glycolytic genes (LDHA, HK2, GLUT1, and PKM2) and knockdown of *HIF1A* in A549 cells had an opposite effect (Figure 7(A)). To validate the function of glycolysis in IAV replication, we further used 2-deoxy-D-glucose (2-DOG) to inhibit glycolysis. Our results showed that glycolysis inhibition enhanced phosphorylation AMPKα and ULK1, reduced p62 levels, increased LC3-II conversion, and finally increased PR8 NP expression (Figure 7(B,C)). Taken together, these results indicate that HIF-1α promotes glycolysis to restrict IAV replication.

### AMPKα-ULK1 signalling-mediated autophagy is required for IAV replication

**Figure 8.**
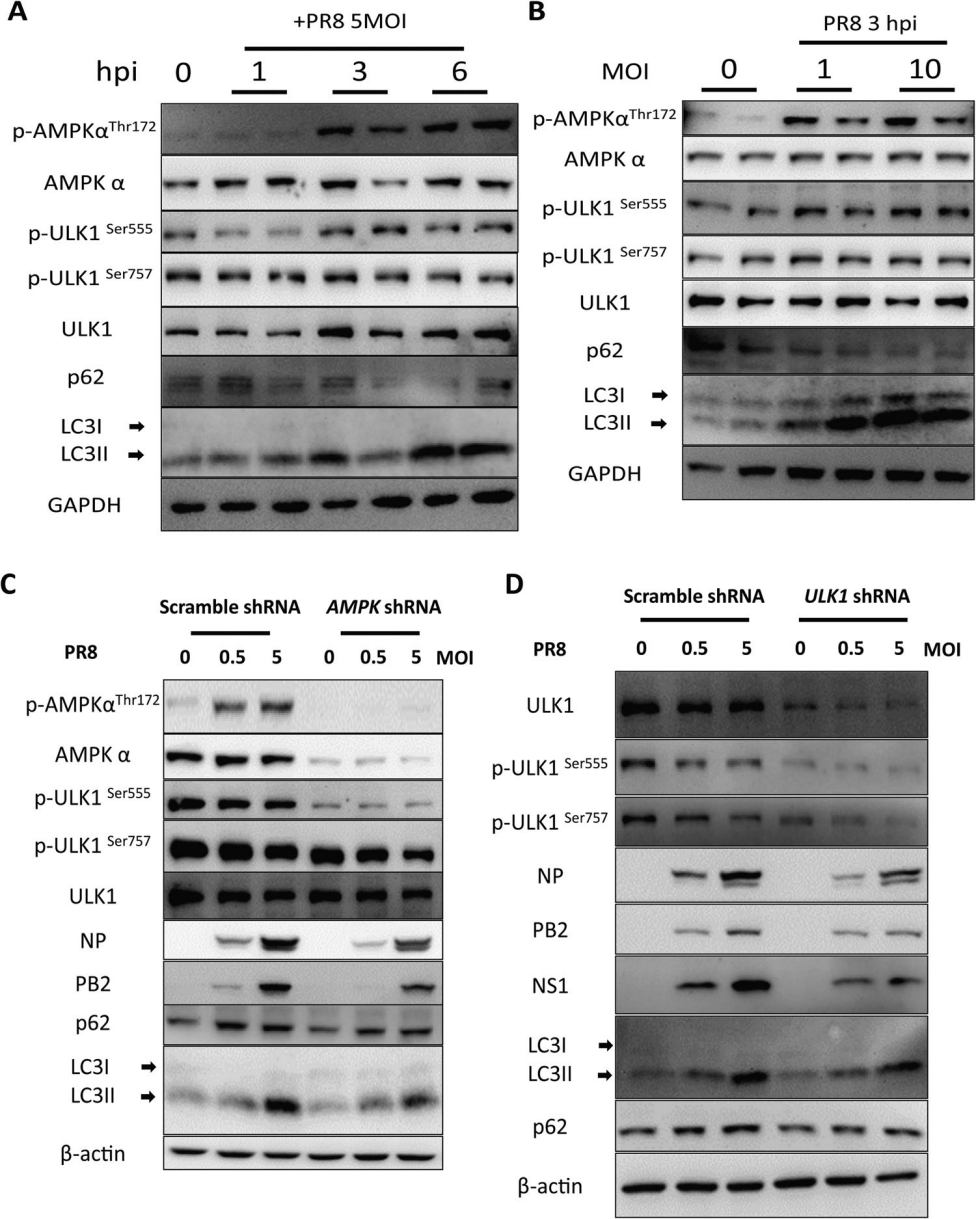
The AMPKα signalling pathway is essential autophagic control of IAV replication. (A) A549 cells were mock-infected or infected with PR8 at MOIs of 5. At 1, 3, and 6 hpi, cells were collected, and cell lysates subjected to Western blot analysis. (B) A549 cells were infected with PR8 at MOIs of 0, 1, and 10, respectively. The cells were collected, and cell lysates were subjected to Western blot analysis at 24 hpi. (C-D) Effect of AMPKα and ULK1 knockdown on IAV replication in A549 cells. A549 cells were infected with pLKO.1 scrambled or shRNA-AMPKα or shRNA-ULK1 lentiviruses, mock-infected or infected with PR8 and then harvested at 24 hpi for Western blot analysis. Results are representative of three independent experiments.

To determine the role of the AMPKα-ULK1 axis in IAV infection and replication, we first tested whether IAV infection activates the AMPKα signalling pathway. Our results showed that IAV infection activated AMPKα-ULK signalling pathway in several hours post-infection but not 24 h post-infection. We found that autophagy is induced during IAV infection (Figure 8(A,B)). To figure out the role of AMPKα in autophagy during IAV infection, we knocked down AMPKα expression in A549 cells by lentivirus-mediated specific shRNA gene silencing. Our results showed that knockdown of AMPKα in A549 cells blocked AMPKα-ULK signalling pathway and decreased autophagy and IAV replication (Figure 8(C)). Similarly, knockdown of ULK1 in A549 cells decreased autophagy and IAV replication (Figure 8(D)). Taken together, these results suggest that AMPKα-ULK1 signalling-mediated autophagy is essential for IAV replication.

## Discussion

In this study, we aimed to elucidate whether HIF-1α regulates IAV replication and the underlying mechanisms. We have found that deficiency of HIF-1α in AEC2 worsens lung inflammation and injury by promoting viral replication during IAV infection. Knockdown of HIF-1α reduces glycolysis pathway, upregulates AMPKα-ULK1 signalling pathway, and enhances autophagy in IAV infected AEC2. To our knowledge, this is the first report demonstrating the role of HIF-1α in IAV replication.

### Requirement for the development of host-factor-directed antiviral drugs

Influenza viruses are a major cause of morbidity and mortality in humans, and influenza A virus in particularly caused pandemic outbreaks in 1918, 1957, 1968 and 2009 [24]. These outbreaks occur with regularity, but the severity was not consistent. Several influenza antiviral drugs have been approved by the US Food and Drug Administration (FDA); oseltamivir, zanamivir, amantadine and rimantadine, these drugs mainly target two of the viral proteins, neuraminidase (NA) and the M2 ion channel protein [25]. However, there is now widespread resistance to these limited viral protein-targeted drugs [26]. Therefore, it is very important to develop new influenza therapies and antiviral drug targets. Identification of host factors in viral replication is becoming an alternative therapeutic strategy to develop new pharmacological targets [13]. Further understanding of host–pathogen interactions in the viral replication life cycle will provide new insight into the development of host-factor-directed antiviral therapies, which could reduce the emergence of viral resistance in therapies.

HIF1 is the major transcriptional regulator that responds to low oxygen tension. As the regulatory subunit of HIF1, HIF-1α is the master regulator of oxygen homeostasis. Besides its role in hypoxia, HIF-1α is involved in many cellular processes. As known, IAV infection-induced ALI led to hypoxia, which further increased the level of HIF-1α in lung tissue. What is the role of the high HIF-1α in the lung during IAV infection, especially in the AEC2? For the influenza virus, the main replicative niche is the lung epithelial cell. Our study focused on the role of HIF-1α in IAV replication in AEC2. We finally uncovered that HIF-1α is a key regulator of IAV replication by interfering with AMPKα-ULK1 signalling mediated autophagy. The results raise the opportunity to utilize HIF-1α signalling in IAV infection as novel therapeutic targets for the development of host-factor-directed antiviral drugs, which needs further studies to determine the possibility.

### Function of glycolysis in IAV infection

HIF-1α is one of the most important regulators of glycolysis. Glycolysis is the main source of energy as cells use this metabolic pathway for ATP generation. Viral infection induces metabolic reprogramming in host cells. Viruses totally rely on host cell machinery to propagate and trigger metabolic reprogramming in host cells to facilitate optimal virus production [27]. It is reported that influenza A virus increased glycolysis by enhancing glucose uptake and lactate production, as well as oxygen consumption rates [27,28]; this is identical to our findings(Figure 7(A)). One study in pediatric patients with respiratory infections also showed there was higher glucose metabolism in the lungs compared to control; oral treatment with BEZ235 (a putative PI3 K/mTOR inhibitor) decreased glycolysis and reduced virus replication which resulted in decreased mortality in the mouse model of influenza infection [29]. This is controversial to our findings in this study. Because oral gavage can’t deliver drugs to AEC2 specifically, it suggests that glycolysis in different cells may have different function in IAV replication.

Glycolysis also plays a critical role in host cell antiviral responses. In pDC, glycolysis could be induced by virus infection and that regulate pDC antiviral functions, including IFN-α production and phenotypic maturation [30]. There is a antagonism between innate antiviral signalling and glycolysis; lactate produced by glycolysis is a natural suppressor of RLR signalling by targeting MAVS, whereas RLR activation suppresses glycolysis by inhibiting hexokinase [31,32]. Here, we show that HIF-1α enhances glycolysis by regulating transcription of glycolytic enzymes and therefore, when HIF-1α is absent, the cellular ATP pool is drastically reduced. Autophagy is a dynamic physiological process that can generate energy and nutrients for cell survival under stressors. This is a feedback response to lower energy level in IAV infection. From this aspect, it is not surprising that HIF-1α depletion increases autophagy as a protective response for the cells during IAV infection.

### Role of HIF-1α and autophagy in AEC2s during IAV infection

The adult lung is a quiescent organ but responds rapidly to injury by activating survival signalling. Hypoxia-inducible factor 1 (HIF-1) plays a key role in the regulation of oxygen homeostasis. In the alveolar regeneration stage after IAV infection, HIF-1α plays a key role in AEC2 differentiation and alveolar repair by driving Notch signaling [22]. An aberrant oxygen environment at birth depletes half of AEC2s in adult mice, which increases the severity of respiratory viral infections later in life [33]. AEC2s function as adult stem cells capable of both self-renewal and differentiation into AEC1s [34]. AEC1s and AEC2s are targets of IAV, and may die during infection. This raises the question of how HIF-1α functions in IAV replication in AEC2.

Our studies show that deficiency of HIF-1α decelerates glycolysis to activate AMPKα-mediated autophagy, which in turn promotes IAV replication inAEC2s.

From autophagy aspect, our study is not the first one to report that autophagy is essential for IAV replication and even the underling mechanism has been clarified clearly [9,35,36]. The novelty of our story is to demonstrate how HIF-1α regulates autophagy in IAV infections. Additionally, we demonstrate that decreased glycolysis due to deficiency of HIF-1α, activates autophagy via AMPKα signalling during IAV infection. AMPKα senses cellular energy status and facilitates autophagy initiation [11,12]. AMPKα activates ULK1, which initiates autophagy by driving the formation of the phagophore. Moreover, activated AMPKα directly phosphorylates ULK1 on Ser 555 resulting in its activation. HIF-1α has been reported to be involved in autophagy under some disease conditions [37,38]. Previous studies reported that the HIF-1α signalling pathway could contribute to autophagy by increasing the expression of BNIP3, which is closely related to autophagy [39,40]. However, our data suggests that, in AEC2, HIF-1α signalling is essential to inhibit autophagy and contribute to control IAV replication. Additional studies are needed to expand to other viruses, which also could utilize autophagy to promote virus replication like IAV.

### HIF-1α and inflammatory response in IAV infection

Recently, the function of HIF-1α in immunity has been extensively studied. For example, HIF-1α is essential for the regulation of key immune effector molecules in myeloid cells and regulates the bactericidal capacity of phagocytes [41,42]. Deficiency of HIF-1α in myeloid cells protects *Escherichia coli* or LPS-induced acute lung injury [43]. Many studies have demonstrated that HIF-1α participates in the cellular response to viral infections [44,45]. For IAV infection, HIF-1α has been verified to regulate the transcription of inflammatory cytokines and neutrophil infiltration into the lung [23,46].

In this study, we demonstrate that deficiency of HIF-1α enhances the replication of IAV by promoting AMPKα-ULK1-mediated autophagy in AEC2. Notably, we and another group both found that HIF-1α is critical for the transcription and production of proinflammatory and antiviral cytokines (Figure 5(D)) [23]. Therefore, HIF-1α is identified as a host restriction factor for IAV replication combined its role in antiviral cytokine expression and autophagy. Although antiviral cytokines expression was reduced in HIF-1α knockdown cell line, in the *in vivo* study, the lung inflammation and virus replication in *Spc-Cre*^+^.*Hif1α*^fl/fl^ mice were significantly higher compared to control *Hif1α*^fl/fl^ mice during IAV infection. This finding suggests that increased antiviral cytokines expression, including *Ifnb*, might not play an important role in restricting viral replication in the AEC2 *Hif1a*-deleted lung. Taken together, we speculated that autophagy might be the key factor that promoted viral replication in the early stage of lung IAV infection in the AEC2 *Hif1a*-deleted lung. As we know proinflammatory or immune cells are the main source of cytokines, the current results might be due to increased cytokine production triggered by enhanced IAV replication in the early stage of IAV infection in *Spc-Cre*^+^.*Hif1α*^fl/fl^ mice. Additional studies are needed to distinguish the different role and function of HIF-1α in autophagy and antiviral immune response during IAV infection.

In conclusion, our study unveils a novel mechanism that HIF-1α signalling controls the replication of IAV by regulating AMPKα-ULK1 signalling-mediated autophagy in AEC2s. This study has provided us new insights into the pathogenicity of IAV. Improved molecular understanding of the role of HIF-1α in IAV replication in relation to glycolysis and autophagy in the host cells may facilitate the development of novel host-factor-directed antiviral therapeutics for influenza virus infection.
